# Noble-Metal-Free MIL-101(Cr)@rGO for Formaldehyde SERS Detection

**DOI:** 10.3390/bios15100703

**Published:** 2025-10-18

**Authors:** Harriet Sonia Nalumansi, Fuwei Pi, Jingkun Li, Guoyong Jiang

**Affiliations:** 1 State Key Laboratory of Food Science and Resources, School of Food Science and Technology, Jiangnan University, Wuxi 214122, China; 6230112906@stu.jiangnan.edu.cn (H.S.N.); 7200112075@stu.jiangnan.edu.cn (J.L.); 7210112090@stu.jiangnan.edu.cn (G.J.); 2 Collaborative Innovation Center of Food Safety and Quality Control in Jiangsu Province, Jiangnan University, Wuxi 214122, China; 3 International Joint Laboratory on Food Safety, Jiangnan University, Wuxi 214122, China

**Keywords:** reduced graphene oxide, SERS, metal organic frameworks, VOCs, formaldehyde

## Abstract

The detection of volatile organic compounds (VOCs) is critical for ensuring food safety, particularly for identifying spoilage gases and food adulterants. Surface Enhanced Raman Spectroscopy (SERS) has traditionally relied on noble metals such as gold and silver for strong electromagnetic enhancement. However, these substrates present challenges in terms of cost, stability, and integration into real-world applications. In this study, we explore a hybrid metal–organic framework (MOF) with reduced graphene oxide (rGO) as a SERS active substrate. The developed material showed a good sensitivity for VOC formaldehyde (FA), easily detectable at peak 1452 cm^−1^ and offering an RSD of 16.95%. Since the substrate did not rely on any noble metals for SERS enhancement, this low cost and easy material could be fine-tuned, creating alternative less-toxic materials for detection in industries such as food safety.

## 1. Introduction

The release of volatile organic compounds (VOCs) and volatile amines containing nitrogen through food spoilage, changes in environmental stature, manufacture of chemicals, and external applications has prompted an increased need for detection and analysis. These compounds are not only a threat to human health, because diseases such as cancer and nerve injuries can be induced through exposure, but also a threat to global environmental safety, affecting food, water, and air sources. The prompt detection of liquid-phase VOCs is critical, as it can prevent undesired emissions, which in turn allows for easier risk management and control. Rudimentary methods such as gas chromatography mass spectroscopy (GC-MS), gas chromatography–Fourier transform infrared spectroscopy (GC-FTIR), gas chromatography–flame ionization detection (GC-FID), high performance liquid chromatography (HPLC), gas chromatography ion mobility spectroscopy (GC-IMS), and cavity ring down spectroscopy (CRDS), have low resolution, high cost of equipment, complex pre-treatment, lack of portability, and require skilled technicians, limiting their application on a large scale [1,2].

In recent years, Surfaced-Enhanced Raman Spectroscopy (SERS) has emerged as a widely used analytical technique in the fields of food, materials, and environmental sciences. Its popularity is due to its powerful ability to conduct label-free chemical identification from the Raman spectra of chemicals, along with abundant enhancement of target molecules adsorbed on plasmonic metals such as gold or silver. Despite these advantages, a reliance on using purely gold and silver has revealed several limitations. These include a lack of specificity, interference from complex real-world samples, and oxidation, which reduce substrate performance and durability [3,4]. In addition, conventional plasmonic materials lack mechanisms for achieving rapid target concentrations, hindering their practical applications. To address these challenges, metal–organic frameworks (MOFs) have emerged as promising materials for SERS-based detection enhancement [5]. Their high surface area [6], adjustable pore size [7,8], low skeleton density, and tunable functional groups make them ideal for the selective adsorption and pre-concentration of specific analytes [8]. For example, gold nano-bipyramids (Au NBPs) were enclosed with Zeolite Imidazolate Framework-8 (ZIF-8) to improve its sensitivity to hydrogen sulfide gas (H_2_S) in the Raman silent region (10–500 and 1800–2800 cm^−1)^, preventing spectral overlap in the Raman fingerprint region (500–1800 cm^−1^) [9].

Graphene, a 2D mono layer material [10], has also been used alongside plasmonic substrates because of its stability [11], ability to quench photoluminescence [12], electro-conductivity, and high charge concentration, contributing to the improvements of the shortcomings traditional substrates possess, as mentioned above [13]. However, due to the fact that its properties are usually studied in combination with plasmonic materials, its individual capabilities in Raman are ignored. As Raman spectrometry relies on both chemical enhancement and electromagnetic enhancement, most research has focused on electromagnetic enhancement, as chemical enhancement is not easily separable from electromagnetic enhancement during analysis [14]. This limits the potential of materials that only possess chemical enhancement to be applied in analysis by themselves. Graphene has been reported to provide chemical enhancement during Raman analysis, which has led to the development of some research on graphene-enhanced Raman spectroscopy (GERS) [15]. The study of GERS has been associated with two principles—first layer effect [16] and Fermi level [4], involving charge transfer—which depends on the arrangement of the target molecules onto graphene layers, thus enhancing Raman scattering. In Fermi level, graphene’s Dirac point is adjusted, either by chemical doping or UV irradiation, which allows for either covalent or non covalent interactions to take place [4]. On the other hand, first layer effect pertains to the very first layer of graphene being reactive and ready to easily transfer electrons between molecules, leading to vibrational modes of the first mono layer [16]. This has made the use of graphene derivatives graphene oxide (GO) and reduced graphene oxide (rGO) common in nanomaterial fabrication. Graphene in combination with MOFS has shown to be another alternative approach to improve the drawbacks of gold and silver when applied for SERS [13]. For example, Liu et al. fabricated an MIL-101(Fe)/GO nanocomposite for discrimination of methyl orange (MO) with an adsorption capacity of 186.20 mg·g^−1^, which was significantly better than MIL-101 (Fe), with a capacity of 117.74 mg·g^−1^ [17]. In this work, GO agglomerated in the system, which allowed for an increase in the number of active sites on the surface, as the Fe^3+^ ions coordinating with organic ligands was avoided, whilst decreasing blockage of GO in the GO/MIL-101 (Fe) system. Through electrostatic forces of attraction, MO could adsorb onto the composite surface through π−π stacking. Duan et al. developed a SERS paper-based chip made of rGO/[Ag (NH_3_)_2_]^+^/ATP for the detection of formaldehyde (FA) and acetaldehyde (AA) in human urine and wine with LOD of 0.15 and 1.3 ng·L^−1^ respectively [18]. In this approach the rGO was employed to fine tune the gaps between silver nanoparticles (AgNPs) leading to improved hotspots that could selectively discriminate FA and AA in complex matrices. Furthermore, Naqvi et al. made a SERS sensor from AgNPs on rGO for Rh-6G,4-MBA and 2,4-DNT detection with enhancement factors of 1.29 × 10^5^, 1.18 × 10^5^, and 6.5 × 10^3^, respectively, showing high sensitivity and homogeneity at ultra-low concentrations of the analytes, making it ideal for on-field detection of molecules such as explosives [19]. In another study, Gil et al. demonstrated that a label-free SERS/graphene-FET platform could detect breast and colorectal cancer exosomes with high sensitivity. This dual system provided both optical and electrical read-outs, reducing detection errors and enabling discrimination between malignant and non-malignant exosomes. Their findings showed the potential of graphene-aided sensors as non-invasive and reliable tools for early cancer detection and diagnosis [20].

Thus, we developed a nanomaterial composite of MIL-101(Cr) and rGO to demonstrate the potential of individual MOF-rGO-based substrates for SERS using a simple method. In our strategy, MIL-101(Cr) particles were synthesized via a traditional hydrothermal reaction and rGO was synthesized via Hummer’s method [21] with a subsequent green chemical reduction process [6,22]. The combination of MIL-101(Cr) and rGO was performed ex situ in the presence of ethanol to form the MIL-101(Cr)@rGO composite. MIL-101(Cr) and rGO were selected as ideal materials because of their ability to initiate charge transfer, which contributes to the chemical enhancement mechanism necessary for SERS [20,23,24]. In comparison to the bare spectra of the target, the Raman intensities were enhanced under 532 nm laser wavelength irradiation.

## 2. Materials and Methods

### 2.1. Materials and Reagents

Pristine graphite powder, concentrated sulfuric acid (H_2_SO_4_), potassium permanganate (KMnO_4_), hydrochloric acid (HCl), sodium hydroxide (NaOH), ascorbic acid (ACS > 99%), hydrochloric acid (HCl (36–38%)), chromium (III) nitrate nonahydrate (Cr(NO_3_)_3_·9H_2_O), terephthalic acid (H_2_BDC), and N, N-dimethylformamide (DMF), hydrogen peroxide (H_2_O_2_), formaldehyde (HCHO, FA, 8–14%), ethanol (EtOH > 99.7%), and 4-aminothiophenol (C_6_H_7_NS, 4ATP, 97%) as well as carbon fiber paper (TGPH060P) were purchased from Sinopharm Chemical Reagent Co., Ltd. (Shanghai, China), InnoChem (Beijing, China) and Aladdin Reagent Co., Ltd. (Shanghai, China) All chemicals were used as received without further treatment. Deionized water was used in all experiments.

### 2.2. Instruments

The size and structure were investigated using transmission electron microscopy (TEM) (FEI Talos F200X G2,Thermo Fisher, Waltham, MA, USA) at an acceleration voltage of 200 kV and scanning electron microscopy (SEM) (German ZEISS Sigma 360, Jena, Germany) at an acceleration voltage of 1 kV. X-ray powder diffraction (XRD) analysis was performed using a Powder X-ray diffractometer (PXRD) (D2 PHASER, Bruker AXS Limited, Karlsruhe, Germany) in the scanning range of = 5–50°, step = 0.05°, scanning rate = 0.5°/min equipped with Cu-k-α (30 kV and 10 mA) to determine the structure and composition of the fabricated material. Fourier-transform infrared spectroscopy (FT-IR) was recorded using a spectroscopy spectrophotometer (IS10, Nicolet, Waltham, MA, USA) All SERS spectra were studied using a Raman microscope spectrometer (DXR2xi, Thermo Fisher, Waltham, MA, USA) (laser excitation wavelength λex = 532 nm, laser power 18–24 mW, objective lens 10X, exposure time 0.100 s, 50 µm slit). Sonication for sample mixing was performed using a high-power numerical control ultrasonic cleaner (KQ-400KDE)

### 2.3. Synthesis of Reduced Graphene Oxide rGO

Reduced graphene oxide (rGO) was obtained by the reduction in graphene oxide using ascorbic acid. Graphene oxide was initially synthesized using Hummer’s method in the absence of sodium nitrite because of its toxicity. The synthesis involved the addition of concentrated sulfuric acid (23 mL) to 1 g of pristine graphene, keeping the mixture in an ice bath at 4 °C, and then stirring at approximately 200 rpm for 1 h. 3 g of potassium permanganate (KMnO_4_) was slowly added to the mixture while keeping the temperature below 4 °C, stirred, and allowed to expand for 1 h. After removing the mixture from the ice bath, it was cooled to room temperature, transferred to an oil bath at 40 °C and stirred for 3 h. After adjusting the temperature to 95 °C for 3 h, 46 mL distilled water was added to the mixture and stirred for 15 min. At 25 °C, 150 mL of ice-cold water was added, followed by a dropwise addition of 5 mL hydrogen peroxide, changing the color of graphene oxide from brown to yellow. Sodium hydroxide (0.2 M) was added to adjust the pH to approximately 6. Ascorbic acid (10 g) dissolved in 100 mL of distilled water was slowly added to the graphene oxide at room temperature. The suspension was reduced at 95 °C for 1 h in an oil bath and the resultant black precipitate was filtered, washed with 0.2 M hydrochloric acid and distilled water was added to neutralize the pH. The filtrate was freeze-dried to obtain the rGO powder.

### 2.4. Synthesis of MIL-101(Cr)

MIL-101(Cr) was synthesized according to Bromberg’s method [25]. Chromium (III) nitrate, 5 mmol, terephthalic acid (5 mmol), and deionized water (20 mL) were sonicated briefly, transferred to a Teflon autoclave, and heated in an oven at 218 °C for 18 h. After cooling, the sample was centrifuged at 10,000 rpm for 5 min (Sorvall ST 40R) and washed with DMF and ethanol. The obtained residue was dried in an oven at 70 °C to form MIL-101(Cr) green powder.

### 2.5. Synthesis of Complex MIL-101(Cr)@rGO

MIL-101(Cr)@rGO was prepared by mixing MIL-101(Cr) and rGO in a ratio of 4:1 (weight) in situ, followed by immersion in ethanol under sonication for 45 min. The final product was left to sit for 12 h to allow adequate binding, washed with ethanol followed by distilled water, and dried at 80 °C in an oven to obtain a powdered complex.

## 3. Results and Discussions

### 3.1. Preparation and Characterization of MIL-101(Cr)@rGO

**Figure 1:**
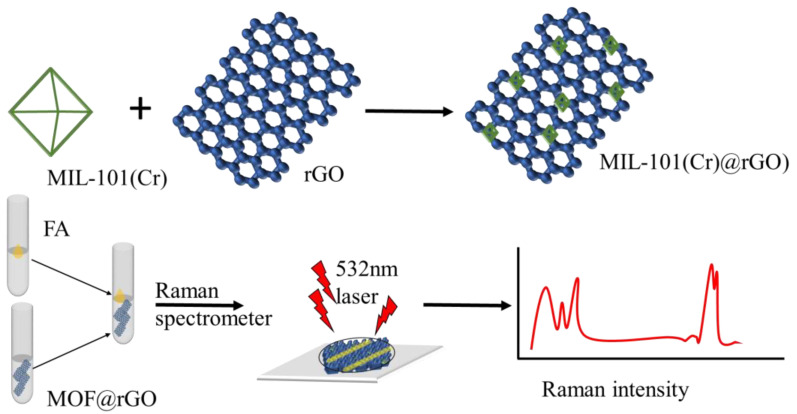
Schematic representation of the fabrication process of MIL-101(Cr) onto rGO for formaldehyde detection.

**Figure 2:**
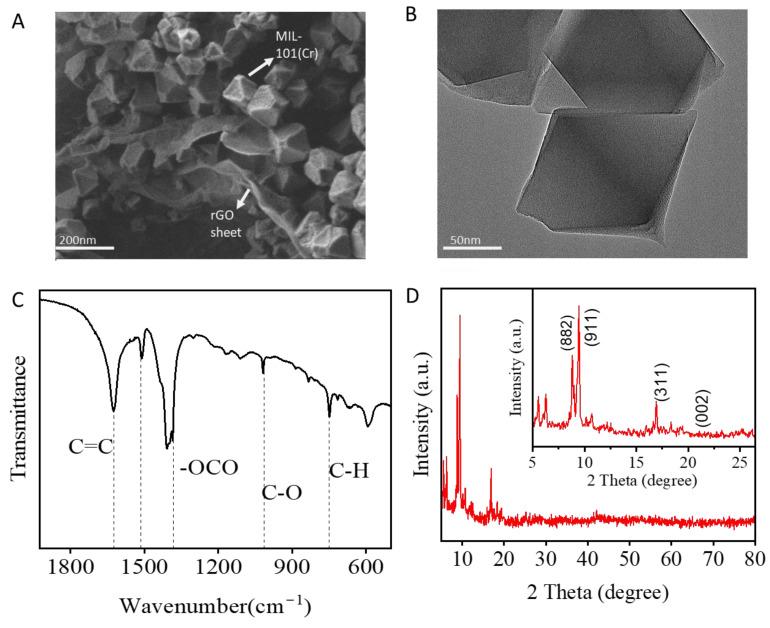
Characterization of MIL-101(Cr)@rGO: SEM and TEM images (**A**,**B**), FTIR and XRD images (**C**,**D**).

Figure 1 shows the steps required to synthesize the MIL-101(Cr)@rGO complex for SERS. The main steps of this process include the pre-synthesis of MIL-101(Cr) and the ex situ loading of rGO. Briefly, MIL-101(Cr) was first synthesized by a microwave-assisted hydrothermal method, to which rGO was loaded to generate improved adsorption sites and dense hotspots. Finally, the MIL-101(Cr)@rGO complex was incubated with the target analyte prior to SERS detection. The morphology and structure of MIL-101(Cr)@rGO were characterized by SEM (Figure 2A) and TEM (Figure 2B), which showed octahedral MIL-101(Cr) crystals embedded with rGO nanosheets.

**Figure 3:**
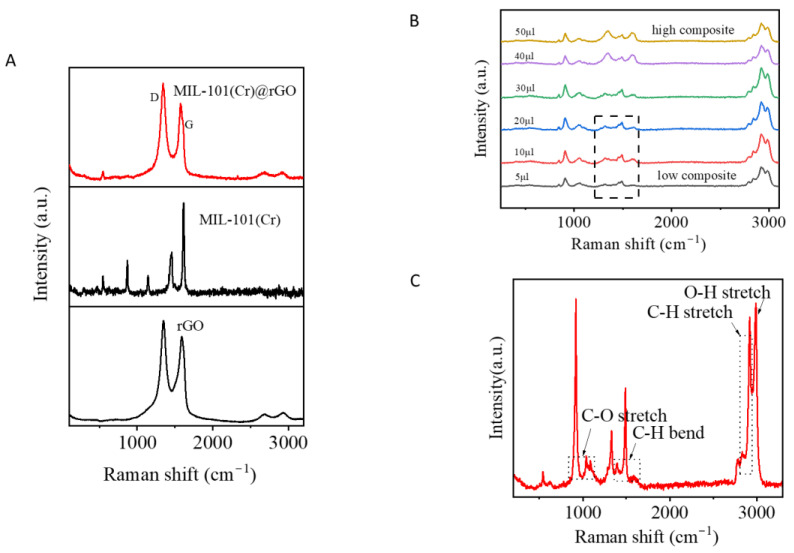
Raman spectra of MIL-101(Cr), rGO, and MIL-101(Cr)@rGO (**A**), quantity distribution effect on the peak intensity at 1452cm^−1^ using concentrated formaldehyde adsorbed onto MIL-101(Cr)@rGO (**B**), concentrated formaldehyde Raman spectra (**C**).

Well-exfoliated but crumpled aggregated rGO sheets were observed in the composite, which is a common result of the oxidation and reduction in graphene oxide. Because rGO mostly consists of single- and few-layer sheets, it can be seen that the MIL-101(Cr) composite acts as an anchor for the sheet. The FTIR spectrum of MIL-101(Cr)@rGO is shown in Figure 2C. The bands around 748 cm^−1^ corresponded to C-H vibration deformation [26]. A distinct peak at approximately 1015 cm^−1^ was associated with C-O stretching, whereas the bands at 1348 cm^−1^ were linked to the -OCO group from the dicarboxylate linker [27,28]. The peaks at 1507 cm^−1^ and 1620 cm^−1^ were attributed to aromatic C=C stretching and the presence of structural water molecules, respectively [26,27,28]. The composite material exhibited well-defined characteristic peaks, and the incorporation of rGO did not alter the formation or the structural integrity of MIL-101(Cr). The XRD Figure 2D pattern of MIL-101(Cr)@rGO is shown. In the MIL-101(Cr)@rGO composite, the characteristic peaks of MIL-101(Cr) at 8.88°, 9.19°, and 17.07°, attributed to the (882), (911), and (311) planes, respectively, and rGO at 21.50°, which corresponds to the (002) plane, were present, confirming that the incorporation of rGO did not alter the crystal structure of MIL-101(Cr) [28,29,30]. The prominent Raman peak around 1600 cm^−1^ could be associated with MIL-101Cr@rGO interactions, potentially arising from π–π stacking between the aromatic rings of the organic linker and the sp^2^ domains of rGO, or from direct coordination between chromium nodes and oxygen-containing groups on rGO. The D band at 1350 cm^−1^ and G band at 1600 cm^−1^, representing defect-induced vibrations and in-plane stretching of graphitic carbon, respectively, confirmed the presence of rGO and offered insights into the degree of disorder and sp^2^ carbon network within the composite [31] Figure 3A.

### 3.2. Optimization

**Figure 4:**
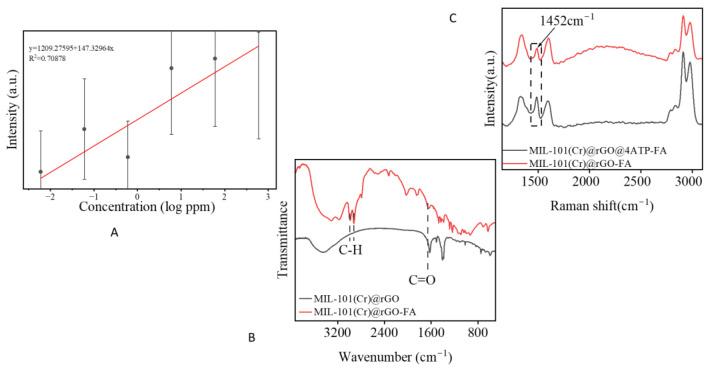
Linear regression plot of varying concentrations(ppm) (2 × 10^−1^ M to 2 × 10^−6^ M) versus formaldehyde. (**A**), Figure showing the effect on 4ATP on formaldehyde peak intensity, (**B**), FTIR spectra showing the effect on formaldehyde adsorbance onto MIL-101(Cr)@rGO (**C**).

To evaluate the potential of reduced graphene oxide as an alternative to conventional plasmonic materials for SERS signal enhancement, initial experiments were carried out using the concentrated formaldehyde solution (1–2 mL) mixed with MIL-101(Cr)@rGO (100–200 µL) and later tested across varying concentrations to assess the signal performance (Table S1). As shown in Figure 3B, increasing the amount of MIL-101(Cr)@rGO led to a corresponding increase in peak intensity, demonstrating that the observed enhancement is strongly dependent on the quantity of the composite present. This suggests that the amount of the composite could influence the charge distribution, thus improving the overall SERS response. While the regression did not yield a perfect linear fit Figure 4A, the correlation coefficient (R^2^ = 0.70) showed a strong relationship, comparable to the values reported in similar SERS studies on formaldehyde detection. (e.g., R^2^ = 0.99 [32], R^2^ = 0.966 [33]). These findings validate the observed results, despite a few experimental deviations. An LOD of 0.36 was achieved from the experiment.

MIL-101(Cr) and rGO were dispersed in ethanol using an ultrasonic cleaner for 45 min at room temperature at five intervals for 9 min to thoroughly mix them in a series of ratios. After optimization and consideration of the factors that each ratio would have on the experiment, such as the Raman signal influence of the bare composite spectrum, 4:1 (weight) was found to be optimal Figure 3A and TEM Figure 2A. The mixture was centrifuged overnight to remove excess ethanol, cleaned, and dried. The reduced graphene oxide formed by ascorbic acid reduction was stable, thus we believe that sonication does not affect the graphene sheets [22,31]. We selected 4-ATP as the ideal Raman probe for the experiment due to its ability to form Schiff base reactions with formaldehyde, which were found to enhance the signal and possibly promote better adsorption of formaldehyde into the composite, as shown in Figure 4B. It is known to be an effective Raman reporter due to its -SH groups and para -NH_2_ functionality, and it can promote charge transfer interaction with aldehyde-formaldehyde thus enhancing the SERS signal [34,35].

### 3.3. Possible Mechanism

According to a comprehensive literature review, graphene and reduced graphene oxide provide significant chemical enhancement, hence the observed signals are attributed to SERS, as the observed spectrum shifts indicate charge transfer between molecules and the substrate. Numerous studies, notably by Shao et al. [36], have demonstrated that Rh-6G Raman spectra on bare SiO_2_ and graphene oxide shows strong G-band signal amplification changes, supporting the charge transfer process. Zhang et al. [4] found that changing the Fermi level of graphene increased the Raman signals through photo-induced charge transfer, proving that graphene substrates actively enable SERS. On the other hand, Ling et al. [16] also created self-assembled PPP monolayers as probe molecules using the Langmuir-Blodgett method, showing that the GERS signal intensity depended on graphene surface closeness, as Raman enhancement was mostly influenced by the primary layer next to graphene, with little beyond the third layer. The results from previous studies confirm the SERS response, which is consistent with our observed results.

Formaldehyde interactions with rGO and MIL-101(Cr) are separately influenced by different processes [37,38,39,40]. For example, Chuang et al. reported that adsorption on rGO occurred mainly through charge transfer and weak physisorption in which the carbonyl oxygen donated electrons into the graphene-π system which can lead to a shift in the G-band and broadening of the D band due to distortion of the sp^2^ network [40]. In contrast, MIL-101(Cr) has been reported to primarily interact with formaldehyde through capture into its porous structure, hydrogen bonding, and polar interactions between the C=O group of formaldehyde and the oxygen framework, while possibly weakly coordinating onto MIL-101(Cr) open sites, as reported by Zhang et al. [37]. Similar findings have been reported previously [38,39].

In the presence of 4ATP, formaldehyde can form an imine once the nitrogen group attacks the electrophilic carbon leading to proton transfer and resulting in a Schiff base [18,34]. However, it could also form hydrogen bonds with the -OH groups in the MIL-101(Cr)@rGO system which aids its capture. Noting that MIL-101(Cr) could posses Lewis’s acid sites [41], this could favor the coordination of the carbonyl formaldehyde group to chromium sites which improves its adsorption onto the substrate surface thereby improving the charge transfer between rGO and formaldehyde Figure 4C. The spectrum demonstrates new absorption peaks of aldehydic C-H stretching in the region 2600–2900 cm^−1^ and carbonyl stretching C=O in regions 1700–1750 cm^−1^ overlapping with MIL-101(Cr)@rGO resulting in sharper peaks confirming the successful adsorption of formaldehyde [42,43,44].The spectral peak shifts also suggest that formaldehyde chemically interacts with the functional groups on MIL-101(Cr)@rGO, supporting a possible chemisorption-based detection mechanism.

### 3.4. Detection of VOC Concentrate Formaldehyde (Raman)

Formaldehyde, a colorless VOC, has been used in industry as a raw material, including but not limited to applications such as cosmetics and construction [45] and is used as a disease biomarker, showing the presence of lung cancer or Alzheimer’s [46,47]. Owing to its degree of reactivity, it exhibits high toxicity and carcinogenic effects, making it a compound of concern that escalates the need for its early detection. The characteristic peaks of formaldehyde are in the region of 3100–3300 cm^−1^ for the O-H stretch as water patches surrounding hydrated oligomers, 2800–3000 cm^−1^ attributed to C-H stretching vibrations, 1720 cm^−1^ likely correspond to the C=O stretching vibration, whereas the region near 1400–1500 cm^−1^ can be associated with C-H bending or deformation modes. Additional peaks in the lower wavenumber range, such as those near 1100 cm^−1^ and 900 cm^−1^, could be linked to C-O stretching and other molecular vibrations specific to formaldehyde [48] Figure 3C.

**Figure 5:**
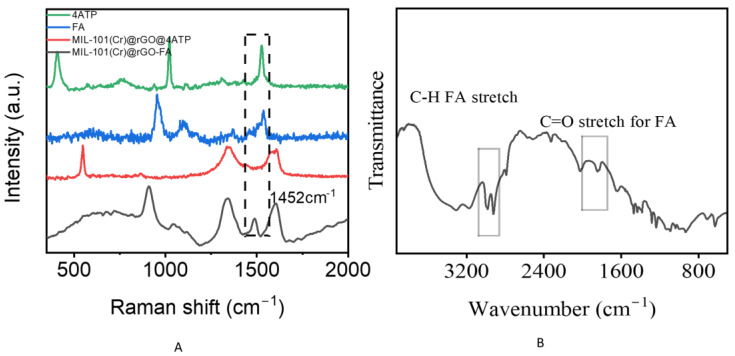
Raman spectra of 4ATP, FA, MIL-101(Cr)@rGO@4ATP, MIL-101(Cr)@rGO-FA, showing detection of formaldehyde(**A**), Mechanism of formaldehyde adsorbance onto MIL-101(Cr)@rGO(B).

Upon exposure of MIL-101(Cr)@rGO to formaldehyde, a formaldehyde peak at 1452 cm^−1^ emerged, resulting in a shift position of the G band from 1600 cm^−1^ to 1680 cm^−1^ with narrowing and sharpening of the peaks. This indicates possible alterations in the electronic structure of rGO due to adsorption, which is consistent with the electron transfer processes between formaldehyde and the rGO surface [40]. The change in the D and G bands suggests an increased density of defects, possibly introduced through covalent interactions or charge-transfer complexes between the aldehyde group of formaldehyde and the oxygen-functionalized sites on rGO Figure 5A.

The strong coupling between MIL-101(Cr) and rGO may have played a role in the detection of formaldehyde. Owing to the syngenetic properties of MIL-101(Cr)@rGO, the high surface area favored the adsorption of formaldehyde onto the surface, acting as an anchor, thus causing a change in the FTIR spectrum, as shown in Figure 5B. The presence of the Raman reporter 4-aminothiophenol (4-ATP) also influenced the spectrum. 4-ATP interacts with formaldehyde through a Schiff base reaction, where the amino group of 4-ATP reacts with the carbonyl group (C=O) of formaldehyde to form a stable imine (Schiff base) linkage. This reaction enabled 4-ATP to adequately bind to the metal centers of the MIL-101Cr@rGO composite, facilitating electronic interactions between the adsorbed formaldehyde, 4-ATP, and the metal–organic framework. These interactions alter the vibrational modes in the Raman spectrum, particularly the peaks associated with the D and G bands of rGO as well as those corresponding to formaldehyde and 4-ATP, reflecting changes in the electronic structure and enhanced charge transfer at the interface. This enhancement of the electronic interactions led to shifts in the peak positions, broadening of bands, and an increase in the overall intensity due to the amplification of the Raman scattering signal through chemical enhancement Figure 4B.

### 3.5. Stability and Reproducibility

Although Ag and Au serve as effective SERS substrates, the reproducibility of the Raman intensity decreases under oxidative conditions. rGO exhibits an extensive sp^2^ hybridized carbon network and fluorescence quenching properties that enhance its chemical and thermal properties, allowing it to serve as either an anchor material to Ag and Au or as an alternative independent SERS substrate [49,50,51,52]. The reproducibility of the Raman signal of MIL-101(Cr)@rGO was assessed based on the intensity of the characteristic peak at 1452 cm^−1^ across eight positions. The calculated RSD was 16.95% showing a moderate signal uniformity Figure S1 (Supporting Information). Although an RSD value below 10% is usually preferred for a highly reproducible SERS platform, values between 10 and 20% are frequently reported for porous and heterogeneous SERS materials. The results show that even though further optimization is required, the developed platform shows potential for practical sensing applications.

The selectivity of MIL-101(Cr)@rGO towards formaldehyde was tested in an equal mixture of acetaldehyde and formaldehyde in equal ratios, as shown in Figure S2. Formaldehyde was detected even when interfering species were introduced.

## 4. Conclusions

Although this research does face certain limitations, it opens up possibilities for new directions. Compared to the extensive usage of noble metals such as gold and silver as SERS substrates, using reduced graphene oxide (rGO) as a standalone platform could exhibit selective sensitivity based on the combination of nanomaterials. However, this method also has a unique advantage. Most SERS studies emphasize the existence of both the electromagnetic mechanism (EM) and chemical enhancement mechanism but only focus on the EM from the plasmons, as it is difficult to separate from the CM, which is not highly visible, thus restricting the applicability of SERS. By shifting the focus to CM-dominant materials and creating rGO-MOF hybrids, this work demonstrates that efficient SERS activity can be achieved without noble metals.

## Data Availability

The original contributions presented in this study are included in the article/Supplementary Material. Further inquiries can be directed to the corresponding author(s).

## Supplementary Materials

The following supporting information can be downloaded at: https://www.mdpi.com/article/10.3390/bios15100703/s1, Figure S1: Graph showing peak intensity variation across eight positions on MIL-101(Cr)@rGO with characteristic band between 1500 and 2000 cm^−1^. Figure S2: Figure showing selectivity towards formaldehyde in acetaldehyde-formaldehyde mixture. Table S1: Table showing varying concentrations of formaldehyde and acetaldehyde with their corresponding ppm values.

## Abbreviations

The following abbreviations are used in this manuscript

VOCS	Volatile organic compounds
SERS	Surface Enhanced Raman Spectroscopy
rGO	Reduced Graphene Oxide
FA	Formaldehyde
MOF	Metal Organic Framework
GERS	Graphene Enhanced Raman Spectroscopy
PPM	parts per million
RSD	relative standard deviation
LOD	Limit of detection
CM	Chemical enhancement
EM	Electromagnetic enhancement
