# Supplementary material for: Noble-Metal-Free MIL-101(Cr)@rGO for Formaldehyde SERS Detection
Source: Biosensors (Basel). 2025 Oct 18;15(10):703. doi: 10.3390/bios15100703 (PMC12564906; doi:10.3390/bios15100703)
Supplement: Supplementary file 1 [file biosensors-15-00703-s001.zip › biosensors-3857322-supplementary.pdf]

Supplementary

Noble-Metal-Free MIL-101(Cr)@rGO for Formaldehyde SERS Detection

Harriet Sonia Nalumansi[1,2,3], Fuwei Pi [1,2,3,*], Jingkun Li [1,2,3] and Guoyong Jiang [1,2,3]

1 State Key Laboratory of Food Science and Resources, School of Food Science and Technology, Jiangnan University, Wuxi 214122, China; 6230112906@stu.jiangnan.edu.cn (N.H.S.); 7200112075@stu.jiangnan.edu.cn (J.L.); 7210112090@stu.jiangnan.edu.cn (G.J.)
2 Collaborative Innovation Center of Food Safety and Quality Control in Jiangsu Province, Jiangnan University, Wuxi 214122, China
3 International Joint Laboratory on Food Safety, Jiangnan University, Wuxi 214122, China
* Correspondence: pifuwei@jiangnan.edu.cn

Received: 20 August 2025
Revised: 25 September 2025
Accepted: 4 October 2025
Published: date

Citation: Sonia, N.H.; Pi, F.; Li, J.; Jiang, G. Noble-Metal-Free MIL-101(Cr)@rGO for Formaldehyde SERS Detection. *Biosensors* **2025**, *15*, x. https://doi.org/10.3390/xxxxx

Copyright: © 2025 by the authors. Submitted for possible open access publication under the terms and conditions of the Creative Commons Attribution (CC BY) license (https://creativecommons.org/licenses/by/4.0/).

Keywords: reduced graphene oxide; SERS; metal organic frameworks; VOCs; formaldehyde

Optimization

To investigate the detection and sensitivity of MIL-101(Cr)@rGO, it was tested at varying concentrations of formaldehyde and the corresponding ppm values were calculated. As a function of concentration, a Raman analysis was performed using a 532 nm excitation source to monitor the intensity of the characteristic formaldehyde peak at 1452 cm^{-1}. The recorded spectra displayed a consistent increase in peak intensity with rising formaldehyde concentration. The linear relationship between formaldehyde concentration and peak intensity (a.u.) was observed within the tested range. The calibration curve yielded the equation

$$I = 147.32964V + 1209.27595$$

Where I is the raman intensity, C is the volume of formaldehyde in μL. The regression coefficient of $R^2 = 0.70878$ and correlation coefficient r = 0.84 show a moderately strong positive correlation Figure S1.

The conversion of formaldehyde concentration to ppm was calculated using the equation:

$$ppm = molarity * molecular\ weight * 1000$$

Given:

Molar mass of formaldehyde = 30.03 g/mol

Molar mass of acetaldehyde = 44.05 g/mol

Table S1. Table showing varying concentrations of formaldehyde with corresponding ppm values

Concentration	ppm Formaldehyde
2 x 10^{-1}M	6006
2 x 10^{-2}M	600.6
2 x 10^{-3}M	60.06
2 x 10^{-4}M	6.006
2 x 10^{-5}M	0.6006
2 x 10^{-6}M	0.06006

Stability, Reproducibility and Selectivity

The reproducibility of the SERS signal was obtained from eight randomly selected positions on MIL-101(Cr)@rGO, with focus on the characteristic peak within the range 1500-2000cm^{-1}. The recorded intensities were

2167.515 a.u.,3157.830 a.u. ,2729.185 a.u. ,2413.239 a.u.,3197.873 a.u. ,3500.000 a.u.,2727.3699 a.u.,2330.764 a.u.

The relative standard deviation (RSD) was calculated using the equation:

$$RSD(\%) = \left(\frac{s}{x}\right) * 100$$

Where s represented the standard deviation of the peak intensities and x represented the mean intensity

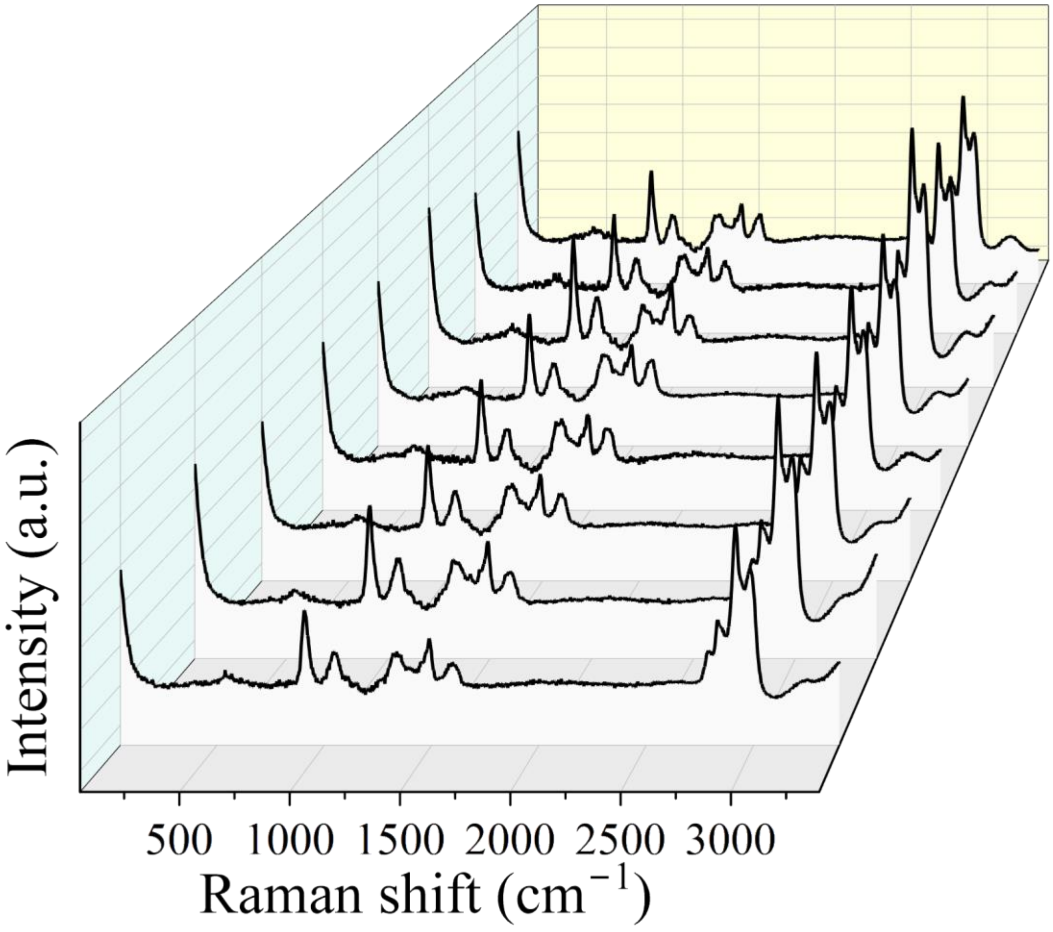

Figure S1. Graph showing peak intensity variation across eight positions on MIL-101(Cr)@rGO with characteristic band between 1500-2000cm^{-1}

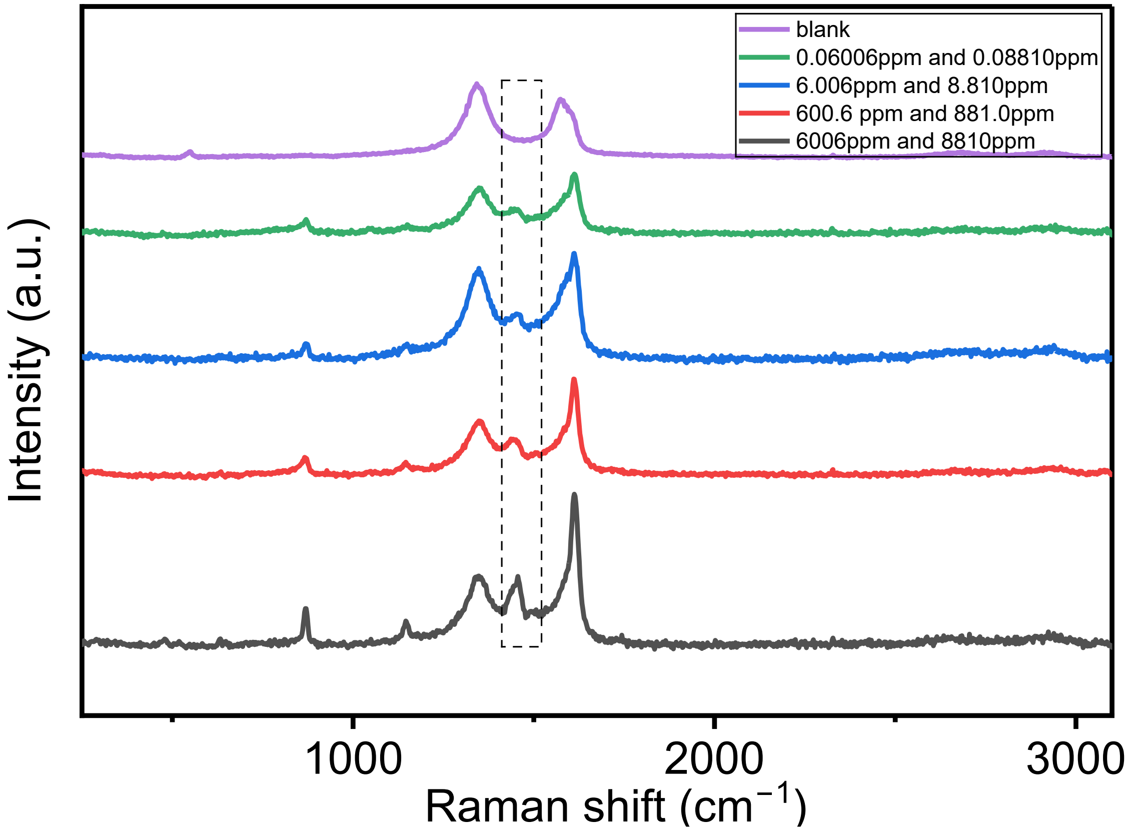

Figure S2. Figure showing selectivity towards formaldehyde in acetaldehyde-formaldehyde mixture.
